# Being there and being with them: the effects of visibility affordance of online short fitness video on users’ intention to cloud fitness

**DOI:** 10.3389/fpsyg.2024.1267502

**Published:** 2024-02-01

**Authors:** Xuewei Chen, Yuyi Zhu, Xinyue Xu

**Affiliations:** ^1^School of Communication, East China University of Political Science and Law, Shanghai, China; ^2^School of Journalism and Communication, Tsinghua University, Beijing, China

**Keywords:** cloud fitness, visibility affordance, social presence, immersion, influencer marketing

## Abstract

**Introduction:**

Cloud fitness is transforming indoor exercise for young people in China. Recent studies have explored the correlation between media use and health-promoting behavior by examining the motivation of individuals and the credibility of influencers. However, the role of media affordance has thus far been largely overlooked. Drawing on the theory of Stimulus-Organism-Response (SOR), the study investigated the indirect effect of visibility affordance on the intention to exercise with fitness influencers in the context of cloud fitness through psychological variables.

**Methods:**

This paper, based on the online survey data (*N* = 456), analyses the effect of visibility affordance on the intention to fitness following with influencers. A moderated parallel mediation model was constructed to examine the relationship among related variables.

**Results:**

The paper draws the following conclusions: (1) Visibility affordance is positively related to the intention to exercise with fitness influencers. (2) Both the sense of social presence and immersion positively mediate the relationship between visibility affordance and the intention to exercise with fitness influencers. (3) The perceived popularity of the influencer positively moderates the relationship between social presence and the intention to exercise with fitness influencers and moderates the mediating role of social presence.

**Discussion:**

Consequently, this study enhances the existing body of knowledge in exercise behavior and health communication literature, and provides practical implications for short video platform, influencers and individuals in promoting healthier behaviors.

## 1 Introduction

Cloud fitness, a rapidly growing form of online fitness, is transforming indoor exercise for young people in China. Cloud fitness refers to the practice of exercising anywhere by following short fitness videos uploaded by fitness influencers on the Internet (known as “*gentiao*” or “*跟跳*” in Chinese), rather than going to a specific gym. We define a fitness-based short video as a clip typically ranging from a few seconds to several minutes, less than 30 min in length, on online platforms, and which introduces home-based fitness programs (Chen and Zhang, 2022). Fitness influencers also share visual information and content related to fitness in short videos, such as showcasing their physiques, demonstrating exercise routines, providing online coaching, and offering free workouts to their followers. Essentially, they act as communicators on social media platforms, disseminating information on sports, fitness, and health to their followers. From this perspective, the visibility affordance of short fitness videos is a determining factor in influencing the fitness intentions of audiences following fitness influencers. Therefore, it is essential to understand how fitness influencers and the visibility affordance of their short videos affect the mental state (e.g., social presence and immersion) and influence fitness intentions. Previous literature has emphasized the positive effects of IT affordances on the purchase intentions of social media commerce consumers (Sun et al., 2019; Tuncer, 2021). However, limited knowledge exists on how to best utilize short videos to promote healthy exercise behaviors in the field of exercise psychology. Song et al. have highlighted that “technology affordances create a positive user experience and facilitate continued use of short-video apps as a source of health information” (p. 2135) (Song et al., 2021). Nevertheless, the affordance theory and understanding of exercise intention in the context of cloud fitness remain under-researched and underdeveloped, deserving more attention. Technology affordance serves as a breakthrough point for exploring the intentions of cloud fitness in the field of exercise psychology. Indeed, within this domain, a review study conducted by Choi et al. (2017) emphasized the importance of environmental factors (e.g., climate, facilities, neighborhood, safety, and home environment) in promoting physical activity. Previous studies have suggested that affordance constitutes a technological environmental stimulus (Sun et al., 2019). In exercise psychology research studies, the role of technology affordance in exercise cannot be neglected, particularly in short videos, which effectively enhance the exercise process.

The emergence of national cloud fitness in China can be attributed to the profound influence of celebrities on online short-video platforms (Tian et al., 2022). These platforms, which include Douyin, Bilibili, and Little Red Book, are home to a diverse array of fitness enthusiasts, comprising fitness instructors, athletes, singers, and actors, who have garnered significant popularity among a wide audience. Notably, Genghong Liu, a renowned Chinese singer, has amassed an impressive following of 70 million fans through his fitness videos on Douyin. Furthermore, German fitness model Pamela Reif boasts over 10.16 million followers, with her online fitness videos on Bilibili garnering a staggering 400 million views, piquing public interest in indoor exercise. According to a recent report published by QuestMobile (2022), the number of internet users in China who follow online fitness key opinion leaders (KOLs), including celebrities and influencers, has reached an astonishing 598 million. This underscores the urgent need to delve into the motivations and intentions of individuals who engage with these fitness enthusiasts on short-video platforms. While previous studies have examined the relationship between credibility, motivation, intention to view fitness videos, and exercise intention in the context of online fitness influencers (Sokolova and Perez, 2021), there is a paucity of published research exploring the impact of visibility affordances, internal states, and the popularity of these influencers on individuals’ fitness intentions. In this article, we aimed to bridge this gap by investigating the influence mechanism and boundary condition between visibility affordances and fitness intention. Specifically, we seek to elucidate how health professionals and short-video providers can effectively harness the power of online short videos to promote healthy exercise lifestyles among their viewers.

Cloud fitness has not only gained popularity on online audio-visual platforms, but it has also notably transformed individuals’ daily exercise routines. This study focuses on cloud fitness as an important phenomenon in this context. Specifically, the goal of this research was to investigate the effects of visibility affordance on fitness decision-making. The research question that this study aimed to answer is as follows: How and under what condition does the visibility affordance of fitness videos on short-video platforms impact the followers’ intention to exercise with fitness influencers? Based on the stimulus-organism-response (S-O-R) theory, we propose a research model to examine the mediating and moderating effects of internal state variables in the relationship between visibility affordance and intention to exercise with fitness influencers. The findings of this study could help health communication practitioners more effectively utilize short videos to promote healthy exercise behavior and encourage physical activity.

## 2 Theoretical framework and research hypotheses

### 2.1 Online fitness and stimulus-organism-response framework

To gain a comprehensive understanding of social media users’ short fitness video watching and exercise with fitness influencers, this study relies on the S-O-R model. The S-O-R framework explains how various environmental stimuli can affect individuals’ behavioral responses by influencing internal states (Mehrabian and Russell, 1974). According to Mehrabian and Russell (1974), this theoretical framework describes a set of organism (O) factors (i.e., cognitive state and affective state) that mediate and thus explain the effects of stimulus (S) factors (e.g., platform characteristics) on their response (R) (e.g., behavioral intentions) (Mehrabian and Russell, 1974; Masrom et al., 2021). As an external or environmental factor, stimulus (S) can change the individual’s internal state and behaviors. Organism (O) is a mediating factor that refers to the internal state or individual’s perception, such as cognitive state and affective state. Response (R) is the consequence of external factors and internal state and represents the individual’s behavioral responses.

Although this model was developed in environmental psychology, many researchers have used this framework to examine individuals’ behavioral responses in different research areas such as store environment, online learning, online social networks, and online video watching (Vieira, 2013; Zhai et al., 2020; Masrom et al., 2021; Wang and Yue, 2022). In the fields of online health and fitness, recent studies also utilizing the S-O-R theory have demonstrated the effect of platform characteristics as environmental stimuli on individuals’ internal reactions and consequent behavioral responses related to fitness applications and community (Elsotouhy et al., 2022; Teng and Bao, 2022; Zhou et al., 2022). However, researchers have paid little attention to applying this framework in short fitness videos. For example, Li (2019) asserted that the S-O-R model can be used in short-video situations. They used the S-O-R model to discuss the impact of video quality and credibility on tourists’ perception of affection and the impact of perception on behavioral intention. Thus, S-O-R is also used as a theoretical framework in our study. According to the S-O-R model, short fitness video users’ fitness intention with fitness influencers can be explained by an environmental and internal path. In the context of the short fitness video, visibility affordance represents the platform environmental “stimuli,” the “organism” is categorized as individuals’ sense of social presence and immersion, and the intention to exercise with fitness influencers represents the “response” in this study. Moreover, these variables were chosen from the previous study (Sun et al., 2019; Tuncer, 2021).

### 2.2 Visibility affordance and fitness behavioral intention

According to the literature, social media affordance includes visibility affordance, persistence affordance, editability affordance, and association affordance (Leonardi et al., 2013). Treem et al. (2020) mentioned that “communication visibility is the root affordance, or possibility for action, for CMC (computer-mediated communication).” A short-video application is an example of a computer-mediated communication platform that enables fitness influencers to share their workout videos. Therefore, visibility is also the root affordance for short-video applications, which constitutes the dimensions of short-video application affordance.

According to visibility research in the field of live streaming commerce (Sun et al., 2019; Tuncer, 2021), we propose that visibility affordance refers to providing ease of access to short fitness videos and visibility of detailed information and instructions concerning the workout action on the short-video platform. It reflects the extent to which short-video platforms present fitness information and instructions from a visual dimension through a short video. In the field of affordance, existing research indicates that visibility affordance is an important component of environmental stimuli within the framework of the S-O-R model (Tuncer, 2021; Xu et al., 2021). Fitness influencers lead the public to exercise online by showing detailed steps in short fitness videos. When the visibility of short fitness videos is higher, the fitness information and skills conveyed by fitness influencers to viewers will be more comprehensive and vivid. While existing studies have extensively discussed the indirect effects of visibility within the S-O-R framework (Leonardi et al., 2013; Tuncer, 2021), a notable absence remains in addressing its direct impact on behavioral intention. Drawing upon the above evidence, visibility affordance can influence individuals’ decisions. Based on the above discussions, we propose the following hypotheses:

*H1*: In the short fitness video, visibility affordance positively predicts the intention to exercise with fitness influencers.

### 2.3 Mediating role of social presence and immersion

Sense of social presence and sense of immersion constitute the internal state of short fitness videos within the S-O-R model. According to previous studies, on the one hand, social presence refers to “the degree to which a person is perceived as a “real person” in mediated communication (p. 151)” (Gunawardena and Zittle, 1997). It is a reflection of the level of the “sense of being with another” (Biocca and Levy, 2013), p. 456, which leads them to perceive it as a sense of “being with them.” On the other hand, the sense of immersion refers to a mental state of “being there” (Zhang et al., 2017), which is “a description of a technology, and describes the extent to which the computer displays are capable of delivering an inclusive, extensive, surrounding and vivid illusion of reality to the senses of a human participant (p. 604)” (Slater and Wilbur, 1997). Oh et al. (2018) have previously indicated that “immersion can be objectively measured by the technological affordances of a medium” (p. 2).

Short fitness video is a high-visibility form of cloud fitness via fitness influencers’ exercising presentations. These short fitness videos can visualize the fitness influencers and their detailed actions of workout to viewers as if they were watching the real person at the offline gymnasium, which leads the viewers to perceive the social presence and immersion. Moreover, the realism and vividness of short fitness videos make it easier to attract viewers and influence individuals’ decisions to follow along with fitness influencers. In the live-streaming commerce field, previous studies showed that a sense of social presence and a sense of immersion mediate the impacts of visibility affordance and behavioral intention (Tuncer, 2021; Xu et al., 2021). However, the roles of social presence and immersion have rarely been explored in previous health and exercise psychology-related literature, and no study has yet investigated the role of these two mechanistic variables in promoting fitness. To address this gap, we are trying to shed light on the indirect mechanism through social presence and immersion. The explanation of these two mechanistic variables contributes to a better understanding of the relationship between visibility affordance and exercise intention in the context of cloud fitness. Due to the above reasons, we propose that social presence and immersion serve as parallel mediators of the relationship between visibility affordance and intention to exercise with fitness influencers. Therefore, in this regard, we proposed the following hypotheses:

*H2*: Both (a) sense of social presence and (b) sense of immersion positively mediate the relationship between visibility affordance and intention to exercise with fitness influencers.

### 2.4 Moderating role of influencer popularity

In the realm of social media influencer marketing, an influencer’s popularity is considered an essential characteristic of their credibility. Follower size, or the number of followers, always serves as a signifier of the influencer’s popularity (De Veirman et al., 2017; Hill et al., 2017). In this article, perceived influencer popularity refers to the popularity of fitness influencers on short-video platforms. Previous research has identified both objective and subjective measures of influencer popularity. Objective indicators cover various parameters, including follower size, number of views, favorites, and comments (Chatzopoulou et al., 2010), while subjective perception relates to an individual’s perceived level of an influencer’s fame, popularity, subscribers, increasing popularity, and comments received on published videos (Lin and Kao, 2010; Ladhari et al., 2020).

Several studies have demonstrated the positive impact perceived influencer popularity has on individuals’ purchasing intentions, attributed to the trustworthiness and credibility of online celebrities (Hill et al., 2017; Ladhari et al., 2020). For instance, Hill et al. (2017) reported higher levels of purchase intentions among individuals who perceive a vlogger as popular than those perceived as less popular. Given these results, we can conclude that influencer popularity also significantly affects individual fitness intention, with higher perceived influencer popularity associated with higher levels of fitness intention. However, despite extensive research into perceived influencer popularity and its impact on individual decisions, the moderating role of an influencer’s level of popularity has yet to be investigated. Therefore, in this study, we propose using perceived influencer popularity as a boundary condition to examine how it moderates the relationship between visibility affordance, social presence, immersion, and exercise intention with influencers.

Previous research has documented that influencer interactivity or interactional tools, such as bullet screens and comments on video platforms, can enhance social presence and immersion (Ladhari et al., 2020; Wei et al., 2022). Online fitness celebrities, who attract a large fan following and receive numerous comments on each published video, are known to be particularly popular. Real-time comments fly across the published video, where viewers can see and view these comments and bullet screens in these high-popularity influencers’ short fitness videos through the platform visibility affordance, highlighting other viewers’ existence and immersing themselves in the video. Considering this, we infer that perceived influencer popularity can moderate the relationship between social presence, immersion, and intention to exercise with fitness influencers and mediate the role of social presence and immersion. Therefore, the following hypotheses are proposed:

*H3*: Perceived influencer’s popularity would amplify the positive relationship between (a) immersion and (b) social presence and intention to exercise with the fitness influencer. Specifically, the association between (a) immersion and (b) social presence and intention to exercise with the fitness influencer is stronger for users with high perceived influencer popularity.

*H4*: Perceived influencer’s popularity moderates the mediating effect of (a) immersion and (b) social presence between visibility affordance and intention to exercise with fitness influencers. Specifically, the stronger the level of perceived influencer’s popularity, the stronger the mediating effect of (a) immersion and (b) social presence.

**Figure 1:**
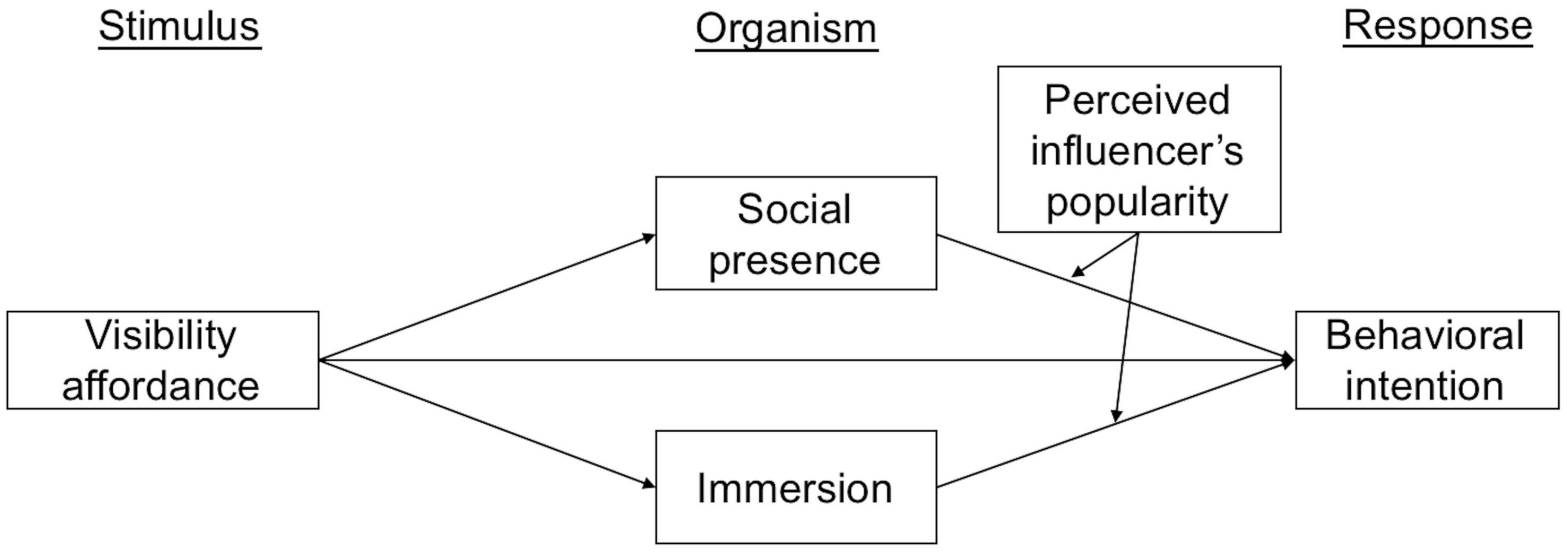
Research model.

To conclude, the research framework is presented in Figure 1.

## 3 Materials and methods

### 3.1 Sampling and data collection

The online questionnaire was distributed and collected from 14 February to 5 March 2023. The data collection protocol was approved by the academic committee of the first authors’ affiliated institution. Participants were recruited from *WeChat*, *Douyin*, *Bilibili*, and *Little Red Book*, which are popular platforms in China that feature professional short videos. All participants were notified with voluntary participation and informed consent before they started the survey. To ensure a representative sample, we targeted users who were active on these platforms and had engaged with short video content in the past month. We used various methods to attract participants, including sharing the link to the questionnaire on social media platforms, and sending private messages to users who had interacted with fitness-related content in the past. Additionally, we provided participants with an incentive to complete the survey by offering a chance to receive a bonus of 5 RMB as compensation. Overall, the recruitment process was designed to reach a diverse group of short fitness video enthusiasts and provide a representative sample for our research.

At the beginning of the questionnaire, we employ a filtering question to ascertain eligible respondents. The question posed is, “Have you recently participated in physical activities by following the guidance of short fitness videos (each video being 30 min or less in duration) featuring fitness influencers?” If their response is affirmative, they proceed to answer the main section of the questionnaire. Conversely, if negative, they discontinue the survey. A total of 481 questionnaires were collected, while 25 of them failed to pass the attention check, resulting in a final sample size of 456. Table 1 presents the sample characteristics. Among all 456 participants, 46.1% (*N* = 210) were males and 53.9% (*N* = 246) were females. The average age of participants was 26 years (SD = 3), 90.6% of them had a bachelor’s degree or higher, and 75.9% of them had RMB 3,000–9,000 yuan monthly income.

**Table 1:** Sample profile (*N* = 456).

Variables	Distribution	Frequency	Percent (%)
Sex	Male	210	46.1
	Female	246	53.9
Age	18–25	241	52.9
(*M* = 26, SD = 3)	26–30	176	38.6
	>30	39	8.6
Education level	Middle school or lower	17	3.7
	High school	26	5.7
	College	190	41.7
	Undergraduate	183	38.6
	Master’s or higher	46	10.3
Monthly income	Less than 3,000 RMB	108	23.7
	3,001–6,000 RMB	112	24.6
	6,001–9,000 RMB	126	27.6
	9,001–12,000 RMB	72	15.8
	12,001–15,000 RMB	17	3.7
	More than 15,001 RMB	21	4.6

### 3.2 Measures

Following rigorous translation and back-translation, all English scales involved in this research were translated into Chinese by the authors. All measurement items are included in Appendix A.

#### 3.2.1 Visibility affordance

The measurement for visibility affordance was adopted from the 4-item Likert-type scale developed by Sun et al. (2019). On a 5-point scale, the participants rated their tendency to describe their feelings when they were watching and selecting short fitness videos online (from 1 = totally disagree to 5 = totally agree). Sample items included the following: “Short fitness videos provide me with detailed instructions related to fitness,” “Short fitness videos make the fitness movement visible to me,” “Short fitness videos make information about how to exercise visible to me,” and “Short fitness videos help me to visualize fitness influencers doing exercises like in the real world.” The reliability of visibility affordance was satisfactory (Cronbach’s alpha = 0.806, *M* = 4.21, SD = 0.66).

#### 3.2.2 Social presence

Items were adapted from Fox and McEwan (2017) to measure individuals’ emotional reactions when they are exercising with fitness videos on a 5-point Likert-type scale (from 1 = totally disagree to 5 = totally agree). Sample items included the following: “Short fitness videos make it seem like the other person (fitness influencers or audience) is present,” “Short fitness videos make it feel like the other person (fitness influencers or audience) I’m exercising with is close by,” and “Short fitness videos make it feel like other people (fitness influencers or audience) are really with me when I exercise” (Cronbach’s alpha = 0.859, *M* = 4.13, SD = 0.79).

#### 3.2.3 Immersion

The 3-item Short Form of Immersion Scale provided by Yim et al. (2017) was used to assess the immersive state when they are exercising with fitness videos. The participants were required to respond to each of the questions about whether they felt deeply engrossed/absorbed/attention-focused or not when they were doing exercises by following the short fitness videos of their favorite fitness influencer by using the relevant range of responses associated with a 5-point scale from 1 = totally disagree to 5 = totally agree (Cronbach’s alpha = 0.870, *M* = 4.24, SD = 0.77).

#### 3.2.4 Perceived influencer’s popularity

Following Ladhari et al. (2020) and Lin and Kao (2010), participants were asked to judge the extent of popularity of their favorite fitness influencer on a 5-point Likert-type scale ranging from 1 = totally disagree to 5 = totally agree, so that the researcher can make sure if the perceived influencer’s popularity is the moderating factor for the popularity of some kinds of short fitness videos. Sample items included the following: “I think my favorite fitness influencer is famous,” “My favorite fitness influencer has a lot of followers,” “The popularity of my favorite fitness influencer is increasing,” and “My favorite fitness influencer has a lot of comments under each published video.” The reliability was satisfactory (Cronbach’s alpha = 0.733, *M* = 4.12, SD = 0.66).

#### 3.2.5 Intention to exercise with fitness influencers

We adopted the 3-item scale used by Venkatesh et al. (2003) to assess intention, which is affected by all the factors above, using a 5-point Likert-type scale ranging from 1 = totally disagree to 5 = totally agree. Sample items are as follows: “I intend to exercise with fitness influencers in the next months,” “I predict I would exercise with fitness influencers in the next months,” and “I plan to exercise with fitness influencers in the next months” (Cronbach’s alpha = 0.788, *M* = 4.11, SD = 0.66).

Additionally, this research also included sex, age, education level, and monthly income as control variables. Sex was assessed as a dichotomous variable (0 = male and 1 = female) and age as a continuous variable (*M* = 26, SD = 3). Education level (middle school or lower = 1, high school = 2, college = 3, undergraduate = 4, master’s or higher = 5) and monthly income (less than 3,000 RMB = 1, 3,001–6,000 RMB = 2, 6,001–9,000 RMB = 3, 6,001–12,000 RMB = 4, 12,001–15,000 RMB = 5, more than 15,000 RMB = 6) were both assessed as ordinal variables.

### 3.3 Analytic procedure

SPSS 25.0 was used for data analysis in this study. We used the SPSS PROCESS macro (model 4 and model 14) to perform the parallel mediation and moderation analysis (Hayes, 2018). We assessed the parallel mediating path and moderating effect using 5,000 bootstrap samples to estimate 95% bias-corrected confidence intervals. First, this study used SPSS 25.0 to perform descriptive statistical analysis for each variable and conduct a common method bias test. Second, we examined the parallel mediation model using the macro PROCESS model 4 in SPSS. Finally, this study used SPSS PROCESS macro model 14 to test the moderated mediation model.

## 4 Results

### 4.1 Preliminary statistics

#### 4.1.1 Common method biases analyses

Harman one-way test was conducted to examine the resulting data for common method bias. The finding revealed that 5 factors in the unrotated factor analysis had eigenvalues greater than 1, and the first factor was 36.944% of the total variance, which was less than the critical value of 40% suggested by Hair et al. (2019). These results indicated that there was no serious common method bias in this study.

#### 4.1.2 Descriptive statistics and correlation analyses

Descriptive statistics and correlations among the study variables are presented in Table 2. The intention to exercise with fitness influencers had a significant positive correlation with visibility affordance (*r* = 0.558, *p* < 0.001), social presence (*r* = 0.530, *p* < 0.001), and immersion (*r* = 0.409, *p* < 0.001).

**Table 2:** Means, standard deviations, and correlations among study variables.

	*M*	SD	1	2	3	4	5
1. Visibility affordance	4.21	0.66	1				
2. Social presence	4.13	0.79	0.504^***^	1			
3. Immersion	4.24	0.77	0.509^***^	0.341^***^	1		
4. Perceived influencer’s popularity	4.12	0.66	0.322^***^	0.247^***^	0.264^***^	1	
5. Behavioral intention	4.11	0.66	0.558^***^	0.530^***^	0.409^***^	0.430^***^	1

*N* = 456. ^***^p < 0.001.

### 4.2 Parallel mediation model

**Figure 2:**
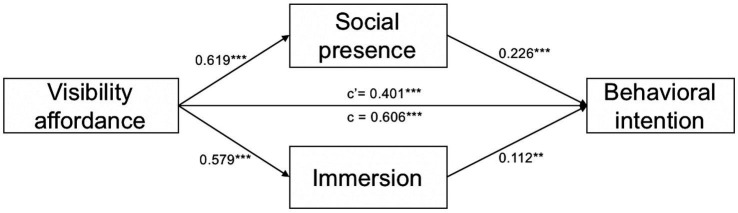
Significant parallel mediation models. ^**^p < 0.01, and ^***^p < 0.001.

This study tested the parallel mediating effect of social presence and immersion between visibility affordance and intention to exercise with fitness influencers based on 5,000 bootstrapping by PROCESS model 4 in SPSS. Table 3 and Figure 2 present the results of regression tests. As predicted by H1, visibility affordance was positively related to the intention to exercise with fitness influencers (*β* = 0.606, SE = 0.037, *t* = 16.298, *p* < 0.001). Consistent with H2a and H2b, visibility affordance was positively associated with social presence (*β* = 0.619, SE = 0.049, *t* = 12.655, *p* < 0.001), and social presence was positively associated with intention to exercise with fitness influencers (*β* = 0.226, SE = 0.034, *t* = 6.669, *p* < 0.001). Similarly, visibility affordance was positively associated with immersion (*β* = 0.579, SE = 0.048, *t* = 12.152, *p* < 0.001), and immersion was positively associated with intention to exercise with fitness influencers (*β* = 0.112, SE = 0.035, *t* = 3.230, *p* < 0.001).

**Table 3:** Multiple regression analysis.

Antecedent	Consequent							
	Model 1 M1 (social presence)		Model 2 M2 (immersion)		Model 3 Y (behavioral intention)		Model 4 Y (behavioral intention)	
	*β*	SE	*β*	SE	*β*	SE	*β*	SE
Sex (female = 1)	0.199^**^	0.075	0.165^*^	0.073	0.105	0.054	0.042	0.052
Age	−0.009	0.011	0.005	0.011	−0.03^***^	0.008	−0.028^***^	0.008
Education level	−0.001	0.032	0.030	0.031	−0.024	0.023	−0.002	0.022
Income per month	0.091^**^	0.027	0.021	0.026	0.148^***^	0.02	0.146^***^	0.019
X (Visibility affordance)	0.619^***^	0.049	0.579^***^	0.048	0.401^***^	0.045	0.363^***^	0.043
M1 (social presence)					0.226^***^	0.034	0.211^***^	0.032
M2 (immersion)					0.112^**^	0.035	0.100^**^	0.033
V (perceived influencer’s popularity)							0.209^***^	0.037
M1 × V							0.224^***^	0.044
M2 × V							0.011	0.055
	*R*^2^ = 0.285		*R*^2^ = 0.282		*R*^2^ = 0.478		R*^2^* = 0.548	
	*F*(5–450) = 35.84, *p* < 0.001		*F*(5–450) = 35.29, *p* < 0.001		*F*(7–448) = 58.49, *p* < 0.001		*F*(10–445) = 54.04, *p* < 0.001	

*N* = 456. ^*^p < 0.05, ^**^p < 0.01, and ^***^p < 0.001.

As shown in Table 4, the indirect effects through social presence [*β* = 0.065, SE = 0.020, 95% CI (0.030, 0.108)] and immersion [*β* = 0.140, SE = 0.032, 95% CI (0.082, 0.207)] were significant. These results showed that both social presence and immersion positively mediated the effect of visibility affordance on the intention to exercise with fitness influencers. Therefore, H2a and H2b were supported. Additionally, the direct effect of visibility affordance on intention to exercise with fitness influencers was significant [*β* = 0.401, SE = 0.045, 95% CI (0.313, 0.488)]; the total indirect effect of visibility affordance on intention to exercise with fitness influencers was also significant [*β* = 0.205, SE = 0.037, 95% CI (0.139, 0.283)]. Thus, social presence and immersion mediated the positive effect of visibility affordance on the intention to exercise with fitness influencers, and the mediation effect accounted for 33.83% (0.205/0.606) of the total effect.

**Table 4:** Direct, indirect, and total effect of parallel mediation.

	Effect	SE	LLCI	ULCI	Proportion of mediating effect
**Direct path**					
Visibility affordance → Behavioral intention	0.401	0.045	0.313	0.488	66.17%
**Indirect path**					
Visibility affordance → Social presence→ Behavioral intention	0.065	0.020	0.030	0.108	10.73%
Visibility affordance → Immersion → Behavioral intention	0.140	0.032	0.082	0.207	23.10%
C1 (Immersion minus social presence)	−0.075	0.038	−0.149	0.001	
Total indirect effect	0.205	0.037	0.139	0.283	33.83%
Total effect	0.606	0.037	0.533	0.679	

### 4.3 Moderated mediation

**Figure 3:**
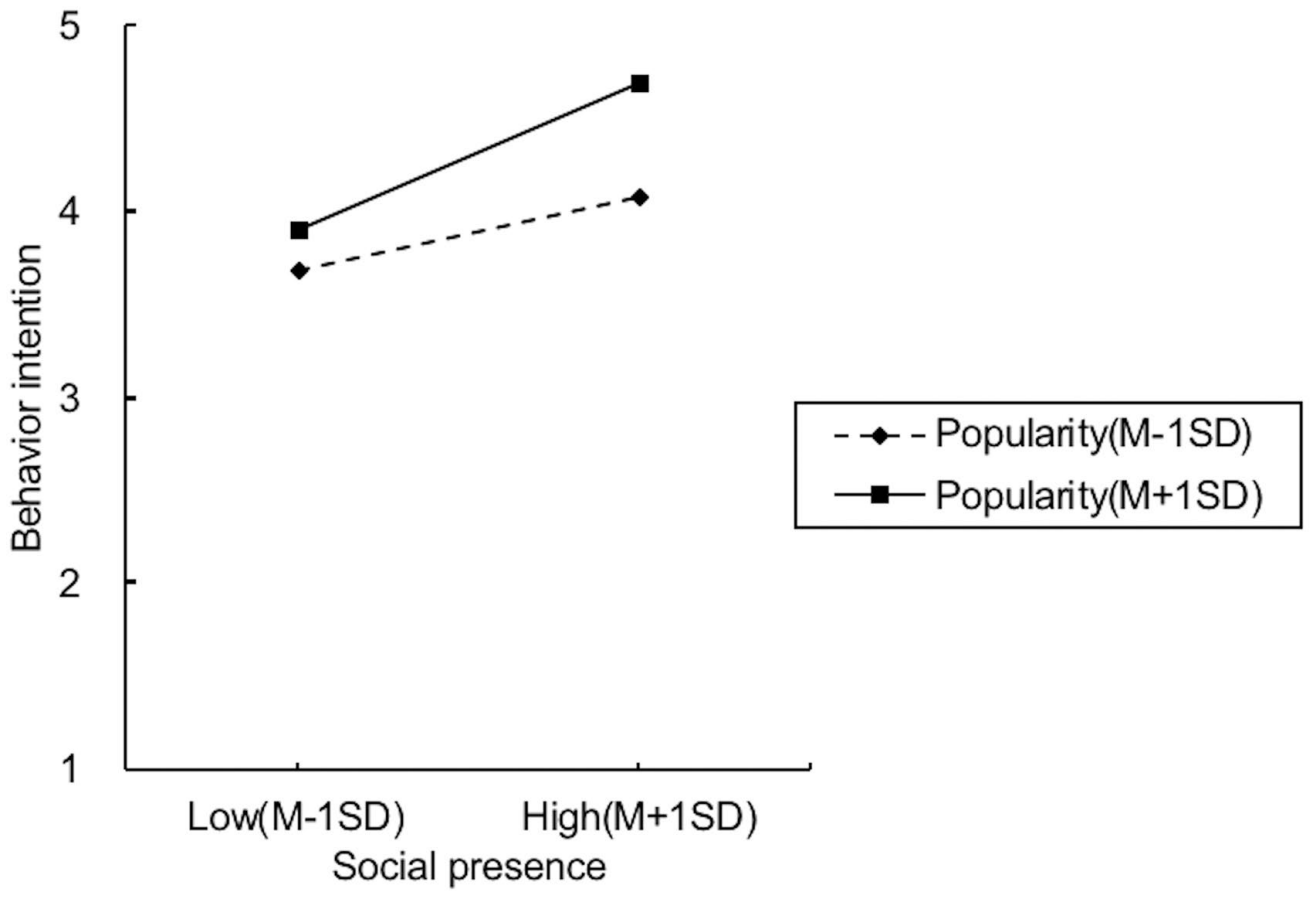
Moderating effect of perceived influencer’s popularity.

We used the PROCESS model 14 to test the moderating effect of perceived influencers’ popularity on the indirect relationship. As shown in Table 3 (model 4), we observed that the interaction of perceived influencer’s popularity with social presence positively predicted intention to exercise with fitness influencers (*β* = 0.224, SE = 0.044, *t* = 5.066, *p* < 0.001), but the interaction of perceived influencer’s popularity with immersion could not predict cloud fitness intention (*β* = 0.011, SE = 0.055, *t* = 0.195, *p* > 0.05). H3b was supported, and H3a was rejected. To demonstrate the moderating effect of perceived influencer popularity, this study conducted a simple slope test as shown in Figure 3. The results of simple slope analysis revealed that, when users’ perceived influencer’s popularity was high (mean + 1SD), the positive effect of social presence on users’ intention to exercise with fitness influencers was 0.502, *p* < 0.001, 95% CI = (0.417, 0.588), and when users’ perceived influencer’s popularity was low (Mean − 1SD), the positive effect of social presence on users’ intention to exercise with fitness influencers was 0.248, *p* < 0.001, 95% CI = (0.165, 0.331). The effect of social presence on the intention to exercise with fitness influencers was greater in participants with more perceived influencer popularity.

**Figure 4:**
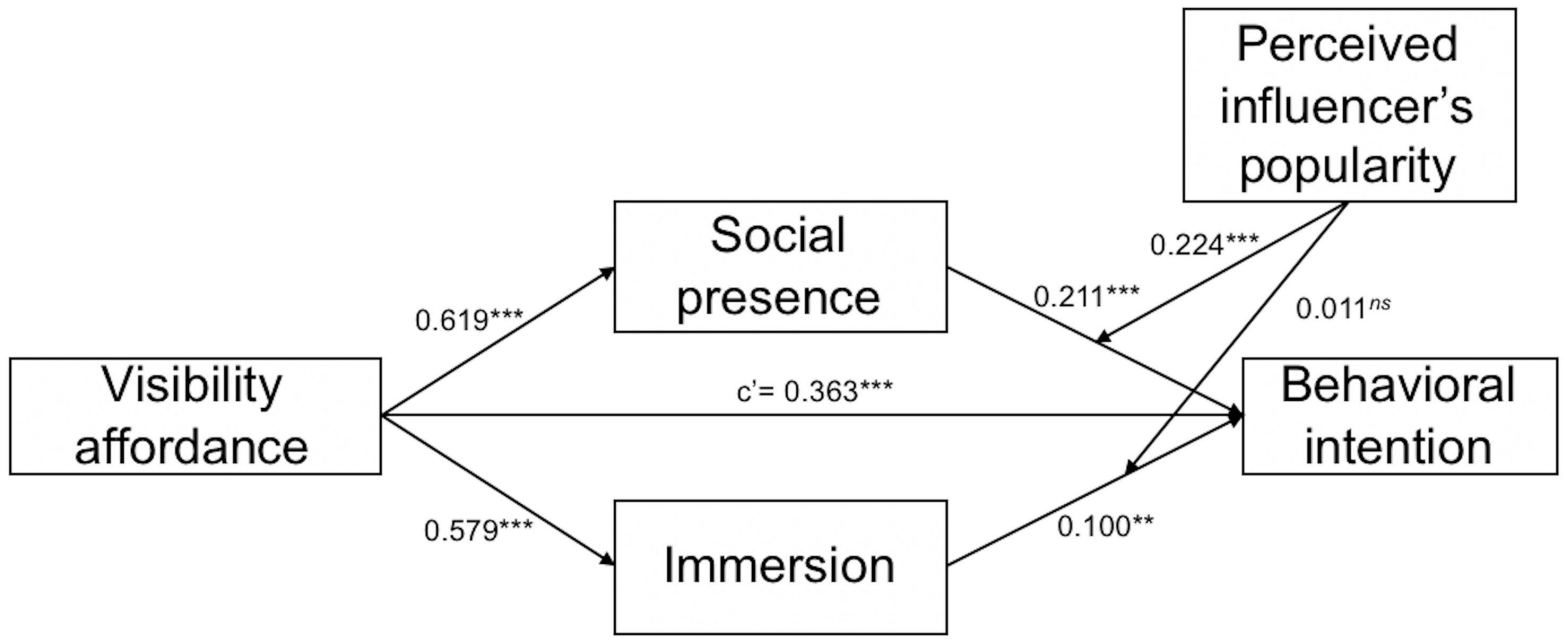
Moderated mediation model. ns ^*^p > 0.05, ^**^p < 0.01, and ^***^p < 0.001.

Furthermore, there is a significant moderated mediation model wherein the perceived influencer’s popularity moderated the indirect effect of visibility affordance on intention to exercise with fitness influencers through social presence [Moderated Mediation Index = 0.139, Boot SE = 0.04, 95% CI = (0.063, 0.222)]. Because the confidence interval did not include zero and had a positive upper bound, we confidently claim that perceived influencer’s popularity positively moderated the mediating effect of social presence, indicating that the indirect effect was stronger for high perceived influencer popularity [*β* = 0.206, Boot SE = 0.031, 95% CI = (0.150, 0.273)]. On the contrary, as the confidence interval includes zero, the perceived influencer’s popularity could not moderate the mediating effect of immersion between the visibility affordance and intention to exercise with fitness influencers [Moderated Mediation Index = 0.006, Boot SE = 0.036, 95% CI = (−0.068, 0.076)]. Thus, H4b was supported, and H4a was rejected. Figure 4 and Table 5 display the results of the moderated mediation analysis.

**Table 5:** Conditional indirect effects of visibility affordance on behavioral intention at values of the moderator.

Moderator value	Conditional indirect effect at means and ± 1SD			
	Effect	Boot SE	Boot LLCI	Boot ULCI
**Social presence**				
Low popularity, −1SD	0.047	0.033	−0.011	0.122
Moderated popularity	0.127	0.022	0.089	0.177
High popularity, +1SD	0.206	0.031	0.150	0.273

## 5 Discussion

### 5.1 Key findings

The current study is an empirical study on short fitness video users’ cloud fitness behavioral intention with fitness influencers and explains this behavioral intention from a S-O-R perspective. To the best of our knowledge, this study is among the first to investigate how and under what condition visibility affordance influences the intention to exercise with fitness influencers in the context of cloud fitness. This current study considered visibility affordance as external stimuli that caused the internal state and predicted their intention to exercise with fitness influencers, and it identified that social presence and immersion represent the consumers’ internal organism. Accordingly, this study aimed to examine how and when visibility affordance facilitates the intention to exercise with fitness influencers. Our results showed that visibility affordance related to social presence, immersion, and intention to exercise with fitness influencers. Moreover, it further discovered the role of social presence and immersion as potential mediators of the relationship between visibility affordance and intention to exercise with fitness influencers. In addition, our results also showed that the moderating effect of influencer popularity on the relationship between social presence and intention to exercise with fitness influencers, and the moderating role of influencer popularity in the indirect association was also confirmed. However, no support was found for the moderating roles of influencer popularity on the relationship between immersion and intention to exercise with fitness influencers. Some discussion of the key findings is provided.

First of all, the results of this study indicated that visibility affordance is a significant antecedent of intention to exercise with fitness influencers. The result confirmed the findings of Sun et al. (2019) and Tuncer (2021) that visibility affordance has significantly positive effects on individuals’ intentions. With a higher level of visibility affordance, short fitness video viewers are more willing to exercise with fitness influencers. Short fitness videos can provide more detailed instructions related to fitness, giving viewers a better fitness experience and a convenient way to follow along on short-video platforms.

Next, we found that social presence and immersion act as mediators to explore the dual-path mechanism of visibility affordance to intention to exercise with fitness influencers. The result verifies the findings of Sun et al. (2019) that social presence and immersion mediate the impacts of visibility affordance on behavioral intention and a higher level of visibility will increase social presence and immersion when they watch online audio-visual content, thereby leading to the consequence of behavioral intention. Therefore, this study proposes that visibility affordance not only increases an individual’s social presence and immersion but also further leads to an increase in their intention to exercise with fitness influencers on short-video platforms. This result enriches the influence mechanism between IT affordance (e.g., visibility) and behavioral intention and further broadens the explanatory power of internal organisms (e.g., social presence and immersion) for fitness follow-along with influencers and other viewers in S-O-R theory.

Third, based on influencer credibility theory, this study introduces the perceived influencer’s popularity as a moderator to explore the potential boundary condition between visibility affordance, social presence, immersion, and intention to exercise with fitness influencers. Previous research has explored the different dimensions of influencer credibility (e.g., homophily, attractiveness, expertise, trustworthiness, and popularity) as antecedents of an individual’s behavior (Ladhari et al., 2020; Sokolova and Perez, 2021; Durau et al., 2022), but attention to considering influencer credibility as a moderator remains insufficient. The moderating role of an influencer’s popularity answers the question: when does visibility affordance foster a viewer’s intention to exercise following along with influencers? On the one hand, the findings suggest that an influencer’s popularity positively moderates the relationship between social presence and intention to exercise with fitness influencers. Specifically, the association between social presence and exercise with fitness influencers is stronger for users with high perceived influencer popularity.

On the other hand, we observe interesting differences in perceived influencer popularity moderating roles in the moderated mediation model. Our results also show that the influencer’s popularity positively moderated the indirect effect. Thus, compared to people with low perceived popularity of the influencer, people with high perceived popularity are likely to believe that the higher visibility affordance inherent in such videos enhances their sense of social presence, and then they would be more actively engaged in cloud fitness. Interestingly, and inconsistent with our hypotheses, perceived influencer popularity could not moderate the relationship between immersion and intention to exercise with fitness influencers, and no support was found for the moderated mediating effects. Potentially, fitness influencers get high attention in short-video platforms and this implies that simply they have high popularity and a large number of followers (Kanaveedu and Kalapurackal, 2022). “Cloud fitness” is a new fitness way that viewers not only see fitness influencers do exercise online, but also they can see bullet screen comments (“*DanMu*” in Chinese) sent by other viewers on screen. It seems like real fitness influencers and real viewers gathering together to keep in shape. As the feeling of social presence refers to a sense of “being with” others (Pimentel et al., 2021), a higher level of visibility affordance constantly drives an individual’s feeling of social presence, and the more willing they were to keep fit following along with fitness influencers. However, immersion is a concept that seems consistent with a sense of “being there” or “flow” (Hamari et al., 2016). Bullet screen comments reflect the comments that fly across the screen, and audiences are easily immersed in the joy of comments, instead of looking at fitness videos. According to Liu and Shi (2022), another possible explanation is that influencer attractiveness, expertise, and interaction can positively predict individuals’ flow experience. Influencer attractiveness, expertise, and interaction may find this set of moderating variables as there seems to be room in heightening individuals’ immersion and fitness intention. Future research seems warranted to examine the moderating role of attractiveness, expertise, and interaction on immersion’s mediated effect. Furthermore, the unique culture of China may exert an influence on these relationships. The collectivist culture prevalent in Eastern countries might foster a stronger sense of social presence and a heightened intention to engage in exercise with influencers, owing to its emphasis on social connectedness and collectivism. Individuals within collectivist cultures prioritize the sense of social presence stemming from interaction. Indeed, Yen and Tu (2011) have also observed that the high-context culture group identifies interactivity as the most pivotal component of online social presence.

### 5.2 Theoretical implications

This study has several theoretical contributions. First and foremost, it significantly enhances the existing body of knowledge in exercise behavior and health communication literature by elucidating the intricate direct and indirect processes underlying fitness intention. Furthermore, it serves to enrich and validate the S-O-R model, which provides a comprehensive framework for understanding an individual’s intention to engage in exercise influenced by environmental stimuli. Specifically, we perceive the elements of “being with them” and “being there” as pivotal facets within this intricate process. In essence, this research delves into the profound impact of the external environment on internal states and behavioral intention, thereby expanding the application and relevance of the S-O-R model in exercise behavior and health communication research. To portray the various components of the S-O-R model, we adopt three sophisticated theoretical constructs: IT affordance, immersion, and social presence. Within this comprehensive framework, visibility is identified as a significant external environmental factor, while social presence and immersion emerge as organic factors of utmost importance.

Second, this study constitutes a valuable addition to the realm of exercise behavior and health communication research, shedding light on the paramount importance of visibility. While the impact of environmental factors, such as natural and indoor settings, on affective states and behavioral intentions during physical activity has been extensively explored within this field (Hösl et al., 2019; Lahart et al., 2019), our study provides a fresh perspective through the lens of “affordance” in the realm of technological environments. This perspective offers insights into the intricate relationship between internal states and individual behavioral intentions. Notably, visibility emerges as a pivotal element within the realm of short-video applications. Our investigation establishes a direct correlation between visibility affordance and behavioral intention. Specifically, we have observed that heightened levels of visibility engender stronger intentions to engage in exercise with fitness influencers. By highlighting the role of visibility affordance in shaping individual fitness intentions within the context of short-video platforms, this study contributes significantly to the existing literature on exercise behavior and health communication.

Third, we have unveiled the intricate psychological mechanisms and boundary conditions that underlie exercise intention. In our investigation, we perceive the act of watching short fitness videos as an internal state that is evoked by the external environment of short-video platforms. To elaborate, we employ two profound psychological variables: immersion and social presence. These variables represent the internal organism of the individual, elucidating their perceptions of “being there” and “being with them” while participating in physical exercise through the guidance of fitness celebrities in short videos. By scrutinizing this intricate relationship through the lenses of social presence and immersion, our study imparts invaluable insights into the psychological mechanisms that drive individuals to engage with and adhere to cloud-based exercise programs. Furthermore, through our examination of influencer popularity, we extend the potential boundary conditions for organisms within the S-O-R model. Specifically, our findings indicate that an influencer’s popularity can enhance the sense of social presence or the profound feeling of “being with them,” consequently reinforcing the correlation between visibility affordance and fitness intention. These significant findings offer profound insights into the intricate dynamics of how and when the visibility affordance of short videos influences viewers’ willingness to engage with the captivating content of fitness influencers.

### 5.3 Practical implications

The positive relationship between visibility affordance and the intention to exercise with fitness influencers highlights the importance of technology affordance in promoting health-promoting behavior. Thus, visibility affordance should be regarded as an important factor for the click and audience ratings of short fitness videos. For instance, this suggests that fitness influencers can effectively use visibility affordances, such as clear visuals and easily accessible information, to enhance their influence on individuals’ exercise intentions. Given the affordability, convenience, and companionship offered by short fitness videos, we anticipate their emergence as a widely embraced and efficacious means of promoting national physical fitness. The low-cost guidance provided by these videos democratizes access to exercise regimes, making fitness more inclusive and accessible to diverse populations. Users can use these insights to make informed choices about the type of online fitness content they consume and the influencers they choose to follow, ultimately enhancing their experience and adherence to health-promoting behaviors.

The mediating roles of social presence and a sense of immersion in the relationship between visibility and affordance also provide implications for promoting national physical fitness. This suggests that individuals’ sense of social presence and immersion can enhance their intention to exercise with fitness influencers when watching fitness videos. In turn, fitness influencers can capitalize on these mediating factors to create a more engaging and immersive viewing experience that encourages individuals to exercise. Short-video providers can leverage the insights from this study to design interfaces and features that accentuate visibility affordance. This could include incorporating high-quality visual elements, real-time interaction capabilities, and tools that enhance the sense of social presence and immersion for users.

The moderating role of the perceived popularity of the influencer underscores the significance of social factors in individuals’ exercise intentions. This suggests that fitness influencers with high perceived popularity may have a greater impact on individuals’ exercise intentions, likely due to their broader social influence and appeal. Consequently, fitness influencers can leverage their popularity to promote exercise intentions by creating engaging content that resonates with their followers. Public health practitioners and policymakers can utilize these findings to develop more effective strategies for promoting physical activity through digital channels. Collaborating with popular fitness influencers and designing campaigns that emphasize visibility affordance, social presence, and immersion could potentially enhance the adoption of health-promoting behaviors among the target audience.

### 5.4 Limitations and future work

This study has some limitations, which could open avenues for future study. First, this study was limited by its cross-sectional design and the results could not explain the cause-effect relationship. Future longitudinal studies and experimental studies could further investigate the causality effects between visibility affordance, social presence, immersion, and behavioral intention to exercise with influencers. Second, the study focused on visibility affordance represented one factor of stimuli (S). Related to this, future studies may explore other dimensions of IT affordances or social affordances, such as accessibility, persistence, bandwidth, engagement, and connectivity on short-video applications (Leonardi, 2011; Argyris and Monu, 2015; Fox and McEwan, 2017). Third, the current study involved young people, but “cloud fitness,” especially fitness with influencers online, is popular among all different ages. Future studies should expand to wider generation cohorts (i.e., X generation, Y generation, and Z generation) to compare the group differences of effect path between the generations and achieve a set of different findings. Finally, we chose a moderating variable (popularity) from influencer credibility theory in the research area of social media influencer marketing. A possible future work could investigate the possible moderating role of body image, body dissatisfaction, and health consciousness. In addition, the length of the short-video clip may also affect its visibility affordance. Future studies could explore the potential impact of video length on visibility affordance and its relationship with social presence, immersion, and behavioral intention to exercise with fitness influencers. This could be done through experimental methods to further understand the effects of video length on these variables.

## Data availability statement

The raw data supporting the conclusions of this article will be made available by the authors, without undue reservation.

## Ethics statement

Ethical review and approval was not required for the study of human participants in accordance with the local legislation and institutional requirements. Written informed consent from the patients/participants was not required to participate in this study in accordance with the national legislation and the institutional requirements.

## Author contributions

XC: Conceptualization, Data curation, Formal analysis, Funding acquisition, Investigation, Software, Writing – original draft. YZ: Methodology, Writing – original draft, Investigation, Writing – review & editing. XX: Conceptualization, Investigation, Supervision, Writing – review & editing.
