# Supplementary material for: Being there and being with them: the effects of visibility affordance of online short fitness video on users’ intention to cloud fitness
Source: Front Psychol. 2024 Feb 1;15:1267502. doi: 10.3389/fpsyg.2024.1267502 (PMC10867133; doi:10.3389/fpsyg.2024.1267502)
Supplement: Supplementary file 1 [file Table_1.DOCX]

Supplementary Material

Being there and being with them: The effects of visibility affordance of online short fitness video on users’ intention to cloud fitness

Xuewei Chen^1^, Yuyi Zhu^1^, Xinyue Xu^2*^

^1^School of Communication, East China University of Political Science and Law, Shanghai, China

^2^School of Journalism and Communication, Tsinghua University, Beijing, China

*** Correspondence:**Xinyue Xu
xuxy21@mails.tsinghua.edu.cn

# Supplementary Figures and Tables

## Supplementary Tables

Following rigorous translation and back-translation, all English scales involved in this research were translated into Chinese by the authors. All measurement items are included in the Appendix A.

**Appendix A.** Instrument of the variables of the standardized factor loading value and scale reliability.

| Variables (sources) | Items | Factor loadings | Scale reliability |
| --- | --- | --- | --- |
| Visibility affordance  (Sun et al., 2019). | Short fitness videos provide me with detailed instructions related to fitness. | .846 | 0.806 |
|  | Short fitness videos make the fitness movement visible to me. | .750 |  |
|  | Short fitness videos make information about how to exercise visible to me. | .817 |  |
|  | Short fitness videos help me to visualize fitness influencer doing exercises like in the real world. | .782 |  |
| Social presence (Fox & Mcewan, 2017) | Short fitness videos make it seem like the other person (fitness influencers or audience) is present. | .851 | 0.859 |
|  | Short fitness videos make it feel like the other person (fitness influencers or audience) I'm exercising with is close by. | .896 |  |
|  | Short fitness videos make it feel like other people (fitness influencers or audience) are really with me when I do exercise. | .816 |  |
| Immersion (Yim et al., 2017) | (I am …… when I do exercises through following the short fitness videos of my favorite fitness influencer.)  Not deeply engrossed–Deeply engrossed | .872 | 0.870 |
|  | Not absorbed–Absorbed | .888 |  |
|  | My attention was not focused–My attention was focused | .881 |  |
| Perceived influencer’s popularity (Ladhari et al., 2020; Lin et al., 2010) | My favorite fitness influencer is famous. | .806 | 0.733 |
|  | My favorite fitness influencer has a lot of followers. | .801 |  |
|  | The popularity of my favorite fitness influencer is increasing. | .661 |  |
|  | My favorite fitness influencer has a lot of comments under each published video. | .707 |  |
| Behavioral intention (Venkatesh et al., 2003) | I intend to exercise with fitness influencers in the next months. | .859 | 0.788 |
|  | I predict I would exercise with fitness influencers in the next months. | .766 |  |
|  | I plan to exercise with fitness influencers in the next months. | .887 |  |
